# Missed opportunities for earlier diagnosis of HIV in British Columbia, Canada: A retrospective cohort study

**DOI:** 10.1371/journal.pone.0214012

**Published:** 2019-03-21

**Authors:** Ni Gusti Ayu Nanditha, Martin St-Jean, Hiwot Tafessu, Silvia A. Guillemi, Mark W. Hull, Michelle Lu, Bonnie Henry, Rolando Barrios, Julio S. G. Montaner, Viviane D. Lima

**Affiliations:** 1 British Columbia Centre for Excellence in HIV/AIDS, Vancouver, Canada; 2 Division of Infectious Diseases, Department of Medicine, Faculty of Medicine, University of British Columbia, Vancouver, Canada; 3 Department of Family Practice, Faculty of Medicine, University of British Columbia, Vancouver, Canada; 4 Division of AIDS, Department of Medicine, Faculty of Medicine, University of British Columbia, Vancouver, Canada; 5 British Columbia Ministry of Health, Victoria, Canada; 6 Vancouver Coastal Health, Vancouver, Canada; ARGENTINA

## Abstract

**Background:**

Late HIV diagnosis is associated with increased AIDS-related morbidity and mortality as well as an increased risk of HIV transmission. In this study, we quantified and characterized missed opportunities for earlier HIV diagnosis in British Columbia (BC), Canada.

**Design:**

Retrospective cohort.

**Methods:**

A missed opportunity was defined as a healthcare encounter due to a clinical manifestation which may be caused by HIV infection, or is frequently present among those with HIV infection, but no HIV diagnosis followed within 30 days. We developed an algorithm to identify missed opportunities within one, three, and five years prior to diagnosis. The algorithm was applied to the BC STOP HIV/AIDS population-based cohort. Eligible individuals were ≥18 years old, and diagnosed from 2001–2014. Multivariable logistic regression identified factors associated with missed opportunities.

**Results:**

Of 2119 individuals, 7%, 12% and 14% had ≥1 missed opportunity during one, three and five years prior to HIV diagnosis, respectively. In all analyses, individuals aged ≥40 years, heterosexuals or people who ever injected drugs, and those residing in Northern health authority had increased odds of experiencing ≥1 missed opportunity. In the three and five-year analysis, individuals with a CD4 count <350 cells/mm^3^ were at higher odds of experiencing ≥1 missed opportunity. Prominent missed opportunities were related to recurrent pneumonia, herpes zoster/shingles among younger individuals, and anemia related to nutritional deficiencies or unspecified cause.

**Conclusions:**

Based on our newly-developed algorithm, this study demonstrated that HIV-diagnosed individuals in BC have experienced several missed opportunities for earlier diagnosis. Specific clinical indicator conditions and population sub-groups at increased risk of experiencing these missed opportunities were identified. Further work is required in order to validate the utility of this proposed algorithm by establishing the sensitivity, specificity, positive and negative predictive values corresponding to the incidence of the clinical indicator conditions among both HIV-diagnosed and HIV-negative populations.

## Introduction

Despite advances in HIV testing programs and improved access to healthcare services, late HIV diagnosis remains a problematic reality in high-resource settings [1–3]. Late presentation to HIV testing has been associated with pre-diagnosis encounters with healthcare providers where an HIV test was indicated, but not ordered; thereby resulting in missed opportunities for earlier HIV detection [4]. Consequences associated with late HIV diagnosis extend across various dimensions of the HIV epidemic: at the individual level, an increased disease burden (e.g., high mortality, risk of hospitalization, and AIDS-defining illness) [5–7]; at the population level, an exacerbated HIV transmission risk [8]; and at a structural level, an amplified healthcare resource utilization and related expenditures [9].

British Columbia (BC), Canada, is the first jurisdiction to implement Treatment as Prevention (TasP), expanded under the Seek and Treat for Optimal Prevention of HIV/AIDS (STOP HIV/AIDS) initiative, which encompasses widespread HIV testing and immediate initiation of free antiretroviral therapy (ART) [10]. Since 2014, provincial HIV testing guidelines have recommended routine testing (i.e., every five years) for individuals aged 18–70 years and annual testing for populations with a higher burden of HIV [11]. HIV testing (nominal and non-nominal), is free of charge for all BC residents. Despite widespread access to HIV testing and the existence of provincial HIV testing guidelines, in 2017, nearly a quarter of diagnosed people living with HIV (PLWH) presented to care with a CD4 count <350 cells/mm^3^ [12]. Taken together, this body of evidence strongly indicates existing opportunities to further optimize the TasP strategy by diagnosing PLWH earlier in the course of HIV infection.

Thus, efforts to understand missed opportunities are crucial to minimize the aforementioned adverse outcomes, and to further optimize TasP in BC and other settings. In this context, insights pertaining to characteristics associated with missed opportunities will be instrumental in achieving the Joint United Nations Programme on HIV/AIDS’ (UNAIDS) target of diagnosing at least 90% and 95% of all PLWH by 2020 and 2030, respectively [13].

In this population-based study, we proposed a case-finding algorithm, based on administrative data, to identify missed opportunities for earlier HIV diagnosis. This algorithm was fundamental in addressing our study objectives, which were: i) to quantify missed opportunities corresponding to a clinical manifestation which may be caused by HIV infection, or is frequently present among those with HIV infection (i.e., clinical indicator conditions) among diagnosed PLWH in BC; and ii) to identify specific clinical indicator conditions and population sub-groups associated with experiencing these missed opportunities. Findings are expected to inform evidence-based recommendations for future interventions, policies, and guidelines surrounding HIV testing.

## Methods

### Study setting

In BC, ART is provided free of charge (no copayments and deductibles) to all HIV-diagnosed BC residents. ART provision has been under the auspices of the BC Centre for Excellence in HIV/AIDS (BC-CfE) Drug Treatment Program (DTP) since 1992. ART eligibility in BC is based on the BC-CfE’s HIV therapeutic guidelines, which have remained generally consistent with those put forward by the International Antiviral Society-USA since 1996 [14].

HIV testing guidelines and practices have evolved in light of sustained advancements in testing technologies resulting in shortened window periods, the availability of point-of-care testing [15], and the emergence of the compelling evidence supporting TasP [16]. Conventionally, testing guidelines were based on the presence of HIV symptoms and known HIV risk factors [17]. In 2010, the BC-CfE set new guidelines recommending healthcare providers to routinely offer an HIV test to sexually active individuals who present to care without having been tested in the year prior, individuals with a history of sexually transmitted infections (STI), and those tested for Hepatitis C, STI, or tuberculosis [17]. In 2014, the Office of the Provincial Health Officer further expanded guidelines by recommending annual testing for those in populations with high HIV burden and testing every five years for everyone else [11].

### Study data

Data were obtained from the STOP HIV/AIDS population-based cohort, which is composed of individual-level longitudinal data on all diagnosed PLWH in the province, by virtue of linkages between various provincial databases and the DTP [10, 18–22]. These aforementioned linkages, along with their corresponding data capture are comprehensively detailed in the S1 File.

### Study design

The eligibility criteria for this retrospective cohort study were as follows: i) ART-naïve individuals aged ≥18 years, ii) who were diagnosed between 1 January 2001 and 31 March 2014, and iii) had a CD4 count measurement within six months. Individuals diagnosed in the acute stage of HIV infection were not considered for missed opportunities given that these infections were detected in a timely manner.

Due to the population-based aspect of this study, HIV testing data is not the exclusive source utilized to ascertain HIV-diagnosed individuals in BC. A considerable number of those HIV-diagnosed have simply never been formally diagnosed via HIV antigen/antibody screening tests (i.e., a confirmed HIV-positive test [10]. Additional data sources of HIV diagnosis play a critical role in supplementing HIV testing data to construct a comprehensive population-based cohort of all HIV-diagnosed individuals.

Thus, HIV diagnosis was ascertained by one of the following validated criterion [23]: a confirmed HIV-positive test, a detectable plasma viral load >50 copies/mL, an HIV-related hospitalization, three HIV-related outpatient care visits, a reported AIDS-defining illness, or ART dispensation. Date of HIV diagnosis was ascertained by the first instance of one of the above-mentioned criteria.

The restriction to ART-naïve individuals was applied to ensure that individuals were accurately classified as a new positive HIV case. Acute HIV infection was defined based on meeting the laboratory criteria (i.e., detection of HIV DNA or RNA by nucleic acid amplification test [NAT], or detection of p24 antigen in the absence of confirmed detection of HIV antibody), or a previous negative or indeterminate HIV test within 180 days of the first confirmed positive HIV test [24].

### Analytical approach and measures

This study consisted of a three-pronged analysis. For all individuals, information pertaining to clinical events identified from healthcare encounters were reviewed for missed opportunities (i.e., the outcome of interest) throughout the following time-frames: i) one year, ii) three years, and iii) five years prior to the HIV diagnosis date. Note that these time-frames were not mutually exclusive, and missed opportunities identified from the five-year analysis also incorporates those identified in the three- and one-year analyses.

This approach strictly served as a sensitivity analysis to account for the considerable individual variability in CD4 counts associated with the natural course of untreated HIV infection. Thus, the inclusion of individuals for each of the analyses was based on the stage of HIV infection at diagnosis. All three analyses comprised of individuals with a CD4 count <500 cells/mm^3^ at HIV diagnosis. Individuals with a CD4 count ≥500 cells/mm^3^ at HIV diagnosis were only included in the one-year analysis in an effort to minimize potential overestimation of the outcome, as these infections are generally of shorter duration. This decision was supported by a large study which estimated that CD4 count depletion reaching the 500 cells/mm^3^ threshold occur on average one year after HIV seroconversion [25]. Other studies opted to examine missed opportunities for HIV diagnosis among individuals diagnosed ≤350 cells/mm^3^ [26, 27], while others have not adhered to any restrictions on the basis of CD4 counts [4, 28].

### Proposed algorithm to identify a missed opportunity

A missed opportunity was defined as a healthcare encounter due to a clinical manifestation which may be caused by HIV infection, or is frequently present among those with HIV infection (i.e., clinical indicator conditions), but no HIV diagnosis followed within a 30-day period. A healthcare encounter was considered to be due to a clinical indicator condition if at least one of the following criterion were met:

1. Medical diagnosis of a condition associated with a prevalence of undiagnosed HIV in the European setting (>0.5%) as specified in the Swiss Federal Office of Public Health 2015 HIV Testing Recommendations [2, 29].
2. Medical diagnosis of an AIDS-defining condition as established by the BCCDC [30];
3. Medical diagnosis of anemia related to nutritional deficiencies or of unspecified cause, which was not mentioned in i) and ii), and identified by expert opinion of two family physicians with >30 years of experience in HIV care (RB & SAG).

These clinical indicator conditions are comprehensively presented in Table 1 and S1 Table. The presence of a clinical indicator condition was ascertained by means of an algorithm using the International Classification of Disease (Ninth and Tenth Revisions) (ICD 9/10) diagnosis codes. This algorithm was applied to the following STOP HIV/AIDS administrative databases: i) the Discharge Abstract Database, which captures diagnostic information on the circumstances of all in-patients discharges, transfers, and deaths, as well as day surgery patients from acute care hospitals across BC; and ii) the Medical Services Plan billing database, which captures diagnostic information related to inpatient and outpatient services provided by physicians and supplementary healthcare practitioners, as well as diagnostic procedures.

**Table 1:** List of clinical indicator conditions utilized in our case-finding algorithm for missed opportunities.

**Source 1: Conditions in which the prevalence of undiagnosed HIV is expected to be >05% in accordance with the Swiss Federal Office of Public Health 2015 HIV Testing Recommendations, as presented in Darling et al**.	**Source 2: BCCDC’s list of AIDS defining illness**
Anal carcinoma/dysplasia*	Bacterial pneumonia, recurrent
Candidaemia	Candidiasis of bronchi, trachea, or lungs
Cervical dysplasia*	Candidiasis of esophagus
Dermatitis/seborrheic rash*	Cervical cancer, invasive
Guillain-Barré syndrome	Coccidioidomycosis (disseminated or extrapulmonary)
Hepatitis B or C (acute or chronic)*	Cryptococcosis (extrapulmonary)
Herpes zoster in an individual <50 years old*	Cryptosporidiosis, chronic intestinal (>1 month)
Invasive pneumococcal disease	Cytomegalovirus disease (not liver, spleen, or nodes)
Lung carcinoma	Cytomegalovirus retinitis (with loss of vision)
Lymphoma*	Encephalopathy, HIV related (dementia)
Mononeuritis	Herpes simplex: chronic ulcers (>1 month)
Multiple sclerosis-like disease	Histoplasmosis, disseminated or extrapulmonary
Oral hairy leucoplakia	Isosporiasis, chronic intestinal (>1 month)
Peripheral neuropathy of unknown origin	Kaposi Sarcoma
Severe or atypical psoriasis	Lymphoma, Burkitt
Sexually transmitted infections*	Lymphoma, immunoblastic (or equivalent term)
Sub-cortical dementia	Lymphoma, primary of brain
Unexplained chronic diarrhea	M.avium/M.kansassi disseminated or extrapulmonary
Unexplained chronic renal impairment	M. tuberculosis (disseminated or extrapulmonary)
Unexplained leuko/thrombocytopenia (>1 month)*	M. tuberculosis (pulmonary)
Unexplained lymphadenopathy	Mycobacterium, other species or unidentified species
Unexplained oral candidiasis	Pneumocystis carinii pneumonia
Unexplained weight loss	Progressive multifocal leukoencephalopathy
Visceral leishmaniasis	Toxoplasmosis of brain
	Wasting syndrome attributed to HIV
	**Source 3. Expert opinion**
	Anemia, unspecified and iron deficiency

Note:

*: The eight indicator diseases previously classified in Sullivan et al. (2013) HIDES (HIV indicator diseases across Europe Study) I.

Given that multiple diagnosis records may correspond to the same unique healthcare encounter, we have employed a set of rules to identify distinct encounters. A healthcare encounter in MSP was considered to be unique if one of the following conditions were fulfilled [31]:

1. If date in which the service was provided by a practitioner was different; or
2. If the practitioner’s specialty was different; or
3. If the location in which the service occurred was different.

An encounter in DAD was considered to be unique from the admission date to the discharge date, including transfers which may have occurred between acute care institutions. In the event that multiple clinical indicator conditions were diagnosed within the same healthcare encounter, only one missed opportunity was counted.

Several data quality control measures have been implemented to ensure coding accuracy [32, 33]. For the DAD, professionally trained coders are responsible for translating the diagnoses from medical charts into ICD-10 codes [34]. The Canadian Institute for Health Information evaluates coding accuracy by conducting reabstraction studies, which involves returning to the original source (e.g., medical charts) and comparing the information with the DAD [32]. These studies have been conducted routinely and yielded favorable results as well as areas for improvement [35]. All acute care facilities in BC are required to report data to the DAD [36]. For MSP, billing records are submitted electronically by a practitioners’ offices to MSP in ICD-9 format. Audits and quality checks for select data fields are subsequently conducted by MSP [18]. It has been demonstrated that these codes are valid at the population level [37]. Nearly 100% of BC residents are covered under MSP [38].

To enhance the rigor of this algorithm, specific restrictions were imposed when constraints were indicated for specific clinical indicator conditions. Recurring conditions required at least two clinical events diagnosed within a 12-month period, while the first clinical event was not considered a missed opportunity. Chronic clinical indicator conditions (i.e., >1 month duration) were considered as a missed opportunity strictly if diagnosed by a specialist. This rule was imposed since administrative data cannot directly capture the duration of a condition; however, conditions diagnosed by a specialist can serve as a proxy measure. For example, conditions with long-standing symptoms may be referred to specialists and are typically associated with potentially lengthy wait times after the initial consultation with a general practitioner [39].

### Explanatory variables

The explanatory variables measured at baseline (i.e., date of HIV diagnosis) included: gender (female and male), age (<30, 30–39, 40–49 and ≥50 years), risk for HIV acquisition (gay, bisexual, and other men who have sex with men [gbMSM], people who have ever injected drugs [PWID], gbMSM/PWID, heterosexual/other, and unknown), CD4 count (<200, 200–349, 350–499, ≥500 cells/mm^3^), calendar year of HIV diagnosis (continuous), BC health authority (Fraser, Interior, Northern, Vancouver Coastal, Vancouver Island, and unknown), and rurality (urban, mixed, rural, and unknown). The health authorities are responsible for the management and delivery of health services in geographically defined areas in BC [40]. Of note, Vancouver Coastal is the largest health authority in BC, caring for >50% of PLWH [41], and where the DTP is located, while Northern is one of our most remote health authorities. Rurality was categorized by classifying local health areas (i.e., 89 provincial health regions aggregated up to the five health authorities) in accordance with their degree of rurality as detailed in S1 File.

### Statistical analysis

Categorical variables were compared using the Fisher’s exact test or the Chi-square test, and continuous variables were compared using the Kruskal-Wallis test [42]. For each of the one-year, three-year, and five-year analyses, an explanatory multivariable logistic regression model was used to model the probability of having 0 versus ≥1 missed opportunity. Model selection was conducted using a backward elimination procedure based on the Akaike Information Criterion (AIC) and Type III p-value [43]. All p‐values are two‐sided, and the level of significance was set at 5%. All analyses were performed SAS version 9.4 (SAS, Cary North CA, USA).

### Ethics

Linkage and usage of administrative databases were approved and performed by data stewards in each collaborating agency and facilitated by the BC Ministry of Health. The University of British Columbia Ethics Review Committee at the St. Paul’s Hospital site provided ethics approval for this study (H18-02208). This study was conducted using strictly anonymized laboratory and administrative databases, and thus informed consent was not required. This study complies with the BC’s Freedom of Information and Protection of Privacy Act.

## Results

### Study population

Of the 2119 individuals in the final analytical sample, the median calendar year of HIV diagnosis was 2007 (25th-75th percentiles [Q1-Q3]: 2003–2011), 82% were male, 53% aged ≥40 years, 75% were either MSM, PWID or both, 56% resided in Vancouver Coastal health authority, 75% resided in an urban area, and 58% were diagnosed with a CD4 count of <350 cells/mm^3^ (Table 2).

**Table 2:** Study population characteristics (N = 2119 individuals).

Variables	Total Individuals n, %
**Sex, n (%)**	
Female	374 (18)
Male	1745 (82)
**Age at HIV diagnosis, n (%)**	
<30	352 (17)
30–39	637 (30)
40–49	641 (30)
≥50	489 (23)
**HIV risk, n (%)**	
gbMSM	943 (45)
PWID	481 (23)
gbMSM/PWID	145 (7)
Heterosexual/Other	520 (25)
Unknown	30 (1)
**CD4 count at diagnosis (cells/mm**^**3**^**), n (%)**	
≥500	504 (24)
350–499	393 (19)
200–349	461 (22)
<200	761 (36)
**Health Authority, n (%)**	
Vancouver Coastal	1178 (56)
Fraser	518 (24)
Interior	126 (6)
Northern	110 (5)
Vancouver Island	162 (8)
Unknown	25 (1)
**Rurality, n (%)**	
Urban	1586 (75)
Mixed	251 (12)
Rural	83 (4)
Unknown	199 (9)
**Number of missed opportunities, n (%)**	
One-year analysis	
0	1977 (93)
≥1	142 (7)
Three-year analysis	
0	1872 (88)
≥1	247 (12)
Five-year analysis	
0	1821 (86)
≥1	298 (14)
**Calendar year of HIV diagnosis, Median (Q1-Q3)**	2007 (2003–2011)

Note: Q1-Q3: 25^th^-75^th^ percentiles; gbMSM: gay, bisexual and other men who have sex with men; PWID: people who have ever injected drugs.

**Fig 1:**
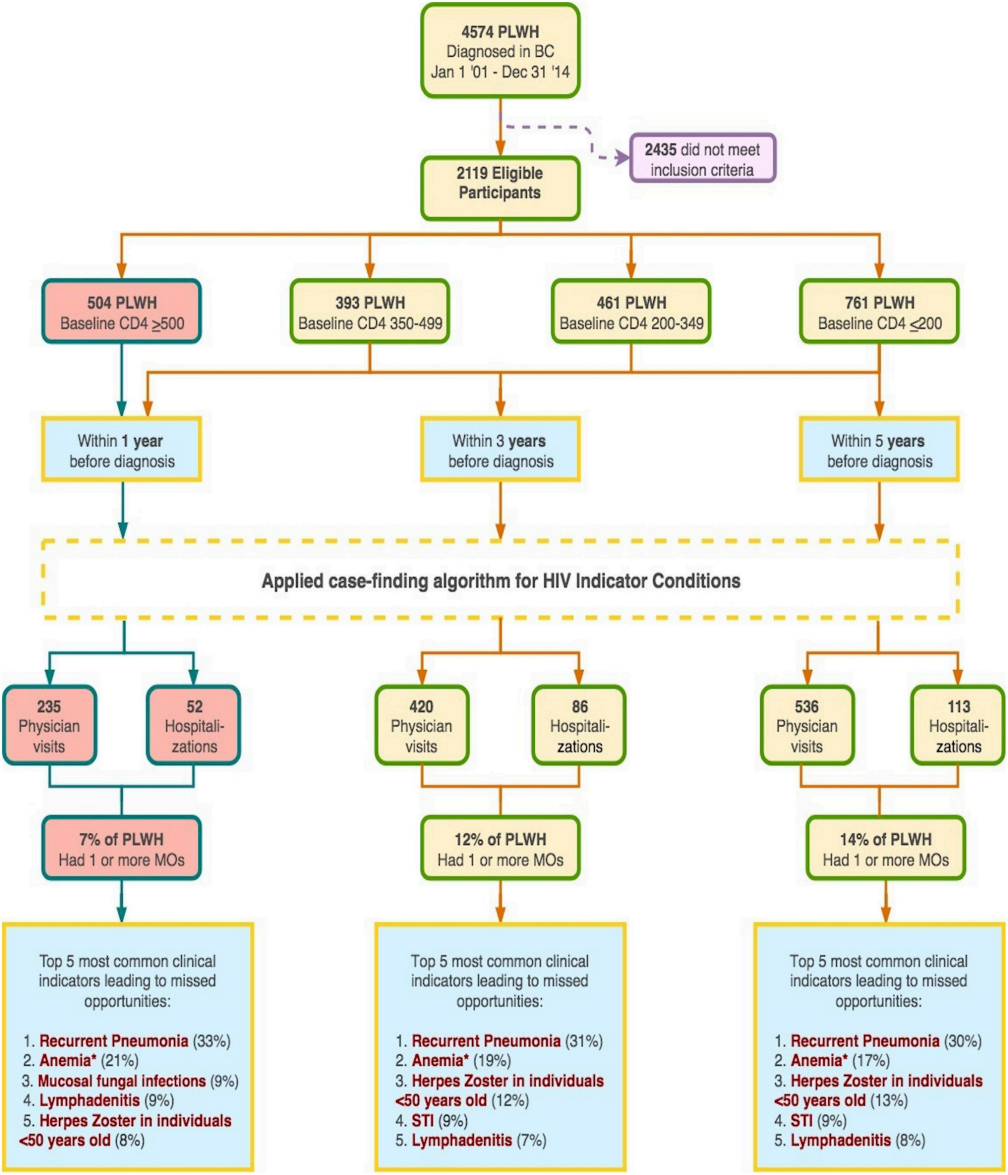
Participants flow chart. PLWH: people living with HIV; STI: sexually transmitted infections; MOs: missed opportunities; *: clinical indicator condition identified by the expert opinion of two family physicians with >30 years of experience in HIV care (RB & SAG).

In the five-year analysis, 298 individuals (14%) recorded a total of 649 unique missed opportunities. In the one-year and three-year analysis, 142 (7%) and 247 (12%) individuals contributed 506 and 287 missed opportunities, respectively (Fig 1). For all three analyses, recurrent pneumonia was the most prominent clinical indicator condition identified as a missed opportunity (33%, 31%, and 30%, respectively), followed by anemia related to iron and other vitamin B12 deficiencies or of unspecified cause (21%, 19%, and 18%, respectively), and herpes zoster/shingles among individuals aged <50 years (8%, 13%, and 13%, respectively). Although not preeminent, sexually transmitted infections, lymphatic disorders and mucosal fungal infections (primarily oral candidiasis) also emerged as important clinical indicator conditions. S2 Table presents the distribution of clinical indicator conditions diagnoses categorized by distinct disease groups, corresponding to the missed opportunities identified in all three analyses.

### Characteristics associated with missed opportunities

#### Bivariable analyses

Consistent across all analyses, having ≥1 missed opportunity was associated with having a CD4 count <200 cells/mm^3^, being older than 50, having a heterosexual/other or PWID HIV acquisition risk, residing in the Northern health authority and in rural areas. An in-depth presentation of these associations, along with their corresponding significance, is reported in Table 3.

**Table 3:** Study population characteristics by analysis, stratified by missed opportunities, (n = 2119 individuals).

Variables	One-Year Analysis			Three-Year Analysis			Five-Year Analysis		
	0 MOs	≥1 MOs	p-value	0 MOs	≥1 MOs	p-value	0 MOs	≥1 MOs	p-value
**Sex, n (%)**			0.3119			0.1604			0.0091
Female	344 (92)	30 (8)		322 (86)	52 (14)		305 (82)	69 (18)	
Male	1633 (94)	112 (6)		1550 (89)	195 (11)		1516 (87)	229 (13)	
**Age at HIV diagnosis, n (%)**			<0.0001			<0.0001			<0.0001
<30	340 (97)	12 (3)		329 (93)	23 (7)		326 (93)	26 (7)	
30–39	613 (96)	24 (4)		591 (93)	46 (7)		577 (91)	60 (9)	
40–49	590 (92)	51 (8)		551 (86)	90 (14)		533 (83)	108 (17)	
≥50	434 (89)	55 (11)		401 (82)	88 (18)		385 (79)	104 (21)	
**HIV risk, n (%)**			<0.0001			<0.0001			<0.0001
gbMSM	900 (95)	43 (5)		864 (92)	79 (8)		849 (90)	94 (10)	
PWID	434 (90)	47 (10)		403 (84)	78 (16)		387 (80)	94 (20)	
gbMSM/PWID	142 (98)	<5 (2)		137 (94)	8 (6)		132 (91)	13 (9)	
Heterosexual/Other	471 (91)	49 (9)		439 (84)	81 (16)		424 (82)	96 (18)	
Unknown	30 (100)	0 (0)		29 (97)	<5 (3)		29 (97)	<5 (3)	
**CD4 count at diagnosis (cells/mm**^**3**^**), n (%)**			0.0002			<0.0001			<0.0001
≥500	479 (95)	25 (5)		479 (95)	25 (5)		479 (95)	25 (5)	
350–499	355 (90)	38 (10)		365 (93)	28 (7)		355 (90)	38 (10)	
200–349	386 (84)	75 (16)		399 (87)	62 (13)		386 (84)	75 (16)	
<200	601 (79)	160 (21)		629 (83)	132 (17)		601 (79)	160 (21)	
**Health Authority, n (%)**			0.0008			0.0001			0.0003
Vancouver Coastal	1114 (95)	64 (5)		1059 (90)	119 (10)		1033 (88)	145 (12)	
Fraser	480 (93)	38 (7)		461 (89)	57 (11)		449 (87)	69 (13)	
Interior	121 (96)	5 (4)		113 (90)	13 (10)		108 (86)	18 (14)	
Northern	92 (84)	18 (16)		84 (76)	26 (24)		81 (74)	29 (26)	
Vancouver Island	146 (90)	16 (10)		131 (81)	31 (19)		127 (78)	35 (22)	
Unknown	24 (96)	<5 (4)		24 (96)	<5 (4)		23 (92)	<5 (8)	
**Rurality, n (%)**			0.0397			0.0123			0.0015
Urban	1486 (94)	100 (6)		1412 (89)	174 (11)		1383 (87)	203 (13)	
Mixed	226 (90)	25 (10)		209 (83)	42 (17)		200 (80)	51 (20)	
Rural	74 (89)	9 (11)		69 (83)	14 (17)		66 (80)	17 (20)	
Unknown	191 (96)	8 (4)		182 (91)	17 (9)		172 (86)	27 (14)	
**Calendar year of HIV diagnosis, Median (Q1-Q3)**			0.7481			0.8746			0.8496
	2007 (2003–2011)	2007 (2004–2011)		2007 (2003–2011)	2007 (2004–2011)		2007 (2003–2011)	2007 (2004–2011)	

Note: MOs: missed opportunities; gbMSM: gay, bisexual and other men who have sex with men; PWID: people who have ever injected drugs; Q1-Q3: 25^th^ percentile -75^th^ percentile].

#### Adjusted multivariable analyses

Factors associated with having ≥1 missed opportunity are comprehensively presented in Table 4 and included:

1. Age: Compared to individuals aged <30 years, those aged ≥50 years had the most elevated odds of having ≥1 missed opportunity for the one-year analysis (adjusted Odds Ratio [aOR]) 2.93; 95% CI 1.52–5.64), three-year analysis (aOR 2.36; 95% CI 1.44–3.89), and the five-year analysis (aOR 2.53; 95% CI 1.58–4.05).
2. Risk of HIV acquisition: Relative to gbMSM, PWID had the highest odds of having ≥1 missed opportunity for the one-year analysis (aOR 1.77; 95% CI 1.12–2.79), three-year analysis (aOR 1.70; 95% CI 1.19–2.43), and the five-year analysis (aOR 1.80; 95% CI 1.29–2.51). Increased odds were also observed for heterosexuals/other compared to gbMSM in each of the analyses.
3. CD4 count at diagnosis: In comparison with individuals diagnosed with a CD4 count ≥500 cell/mm^3^, those diagnosed with a CD4 count <200 cells/mm^3^ had the greatest odds of having ≥1 missed opportunity for the three-year analysis (aOR 3.18; 95%CI 2.01–5.01) and the five-year analysis (aOR 4.07; 95%CI 2.60–6.37
4. Health authority: Relative to individuals residing in Vancouver Coastal health authority, those residing in the Northern health authority had the highest odds of having ≥1 missed opportunity for the one-year analysis (aOR 2.41; 95%CI 1.32–4.41), three-year analysis (aOR 1.94; 95%CI 1.16–3.25), and five-year analysis (aOR 1.78; 95%CI 1.08–2.92).

**Table 4:** Multivariable logistic regression models for factors associated with ≥1 missed opportunities.

Variables	One-Year Analysis aOR (95% CI)	Three-Year Analysis aOR (95% CI)	Five-Year Analysis aOR (95% CI)
**Sex**	NS	NS	NS
Female			
Male			
**Age at HIV diagnosis**			
<30	1.00 (REF)	1.00 (REF)	1.00 (REF)
30–39	1.03 (0.51–2.11)	0.98 (0.58–1.66)	1.14 (0.70–1.86)
40–49	2.10 (1.09–4.05)	1.85 (1.13–3.03)	1.99 (1.25–3.17)
≥50	2.93 (1.52–5.64)	2.36 (1.44–3.89)	2.53 (1.58–4.05)
**HIV risk**			
gbMSM	1.00 (REF)	1.00 (REF)	1.00 (REF)
PWID	1.77 (1.12–2.79)	1.70 (1.19–2.43)	1.80 (1.29–2.51)
gbMSM/PWID	0.44 (0.13–1.44)	0.62 (0.29–1.33)	0.87 (0.47–1.62)
Heterosexual/Other	1.67 (1.07–2.61)	1.56 (1.10–2.22)	1.58 (1.14–2.20)
Unknown	NA	0.41 (0.05–3.12)	0.36 (0.05–2.70)
**CD4 count at diagnosis (cells/mm**^**3**^**)**			
≥500	1.00 (REF)	1.00 (REF)	1.00 (REF)
350–499	0.64 (0.32–1.27)	1.44 (0.82–2.53)	2.03 (1.20–3.45)
200–349	1.14 (0.65–1.99)	2.60 (1.59–4.24)	3.31 (2.05–5.34)
<200	1.51 (0.92–2.45)	3.18 (2.01–5.01)	4.07 (2.60–6.37)
**Health Authority**			
Vancouver Coastal	1.00 (REF)	1.00 (REF)	1.00 (REF)
Fraser	1.22 (0.80–1.88)	0.97 (0.69–1.38)	0.97 (0.70–1.34)
Interior	0.55 (0.21–1.41)	0.77 (0.41–1.43)	0.88 (0.51–1.51)
Northern	2.41 (1.32–4.41)	1.94 (1.16–3.25)	1.78 (1.08–2.92)
Vancouver Island	1.43 (0.79–2.60)	1.53 (0.97–2.42)	1.41 (0.92–2.18)
Unknown	0.56 (0.07–4.30)	0.30 (0.04–2.31)	0.52 (0.12–2.34)
**Rurality**	NS	NS	NS
Urban			
Mixed			
Rural			
Unknown			
**Calendar year of diagnosis**	NS	NS	NS

Note: aOR: adjusted odds ratio; CI: confidence interval; gbMSM: gay, bisexual and other men who have sex with men; PWID: people who have ever injected drugs; REF: reference; NS: not selected; NA: not applicable due to no count in the specified category.

## Discussion

Findings from this population-based cohort study underscored missed opportunities for earlier HIV diagnosis in BC using a newly-developed case-finding algorithm based on ICD 9/10 codes for clinical indicator conditions. This study demonstrated that despite a setting where access to healthcare is unrestricted, opportunities for earlier HIV diagnoses remain; between 7%-14% of individuals experienced ≥1 missed opportunity. The most prominent missed opportunities were related to diagnoses of recurrent pneumonia, anemia related to nutritional deficiencies or of unspecified cause, and herpes zoster/shingles among individuals aged <50 years.

Our results complement the growing body of evidence indicating that individuals in high-resource settings continue to be susceptible to missed opportunities [4, 28, 44, 45]. In West Scotland and Italy, 26% and 29% of individuals had ≥1 clinical indicator condition prior to their HIV diagnosis ever, respectively [4, 28]. In the United States and Switzerland, 22% and 44% of individuals were found to have ≥1 HIV indicator condition within five years prior to HIV diagnosis, correspondingly [44, 45]. The relatively low proportion of missed opportunities in BC may be reflective of rigorous HIV prevention, testing and educational outreach programs in this province [46]. However, readers should be aware that differences exist in the definitions of missed opportunities among studies.

Current HIV testing guidelines acknowledge that HIV can have a range of non-specific presentations and recommend that healthcare providers include HIV in the differential diagnosis, irrespective of identified risk for HIV acquisition. This includes when an individual exhibits symptoms that warrant laboratory investigation or symptoms associated with HIV infection or immune compromise. Our findings complement the aforementioned recommendation by underscoring specific indicator conditions in which missed opportunities were most prevalent (i.e., recurrent pneumonia, anemia related to nutritional deficiencies or of unspecified cause, and herpes zoster/shingles among individuals aged <50 years). These conditions constituted 59%-69% of the missed opportunities, thus should particularly be on the forefront of clinical investigation when considering HIV in the differential diagnosis.

Findings from this study also inform healthcare providers and policy makers with important evidence necessary to implement targeted interventions aimed at improving HIV screening among the identified population sub-groups at increased risk of experiencing missed opportunities. The overarching theme appears to put forward a two-tiered issue: i) need to further emphasize underserved sub-populations, which are disproportionately impacted by stigma and geographical barriers to healthcare access [47, 48]; and ii) need to target individuals from population sub-groups not traditionally associated high HIV burden, which may be inaccurately presumed to be at low risk for HIV by healthcare providers [49, 50]. From a population perspective, these findings illustrate the need to provide further education for health care providers regarding universal testing.

While addressing missed opportunities for earlier diagnosis through increased testing in healthcare settings is a valuable strategy in reducing late diagnosis, individuals who do not present to care (e.g., asymptomatic individuals, those experiencing barriers to care) continue to be overlooked and contribute to late diagnosis in the absence of complementary interventions [50].

There are some limitations to consider. First, although our case-finding algorithm for identifying missed opportunities using ICD 9/10 codes has not yet been validated, thus potentially limiting the extrapolation of the results, it was developed on the basis of established clinical indicator conditions from the literature and informed by experts in the fields of HIV epidemiology and medical care. Second, although a number of clinical indicator conditions were selected based on having a prevalence of undiagnosed HIV >0.5% in the European context, the overall trends in HIV prevalence are largely homogeneous among high-income countries of North America and Western Europe [51]. Third, due to the requirement of an HIV diagnosis for entry into the STOP HIV/AIDS cohort, we were unable to assess the incidence of these clinical indicator conditions among those not HIV-diagnosed. However, case-control studies demonstrated that several of these clinical indicator conditions had a higher incidence among HIV cases compared to matched HIV-negative controls [52–54]. Fourth, HIV negative testing data did not include screening tests from Vancouver Island health authority (<5% of all screening tests), or those performed on a non-nominal basis [55]. Point-of-care testing data was also not available, though it only consisted of a small proportion of all tests performed in BC (<5%) [56]. Fifth, the STOP HIV/AIDS cohort does include data from the National Ambulatory Care Reporting System, rendering us unable to identify missed opportunities occurring during emergency room visits. Sixth, the STOP HIV/AIDS cohort data capture is restricted to March 2014; future analyses will assess missed opportunities after the revision of the provincial HIV testing guidelines in 2014. Finally, although healthcare administrative data are not collected for the purposes of research and are susceptible to misclassification and coding errors, they represent an important source of information for evidence-based research.

In conclusion, based on our newly-developed algorithm, this study demonstrated that HIV-diagnosed individuals in BC have experienced several missed opportunities for earlier diagnosis. Specific clinical indicator conditions and population sub-groups at increased risk of experiencing these missed opportunities were identified. Further work is required in order to validate the utility of this proposed algorithm by establishing the sensitivity, specificity, positive and negative predictive values corresponding to the incidence of the clinical indicator conditions among both HIV-diagnosed and HIV-negative populations.

## Supporting information

S1 File Methodological supporting information. Additional methodological information pertaining to the data utilized in this study.
(DOCX)

S1 Table List of the International Classification of Diseases (Ninth and Tenth Revisions) diagnosis codes selected for the case-finding algorithm for clinical indicator conditions. Note: ICD 9: International Classification of Diseases (Ninth Revisions); ICD 10: International Classification of Diseases (Tenth Revisions).
(DOCX)

S2 Table Diagnoses of clinical indicator conditions categorized by distinct disease groups for all three analyses. (A) five-year analysis, (B) three-year analysis, and (C) one-year analysis. Note: Given that multiple clinical indicator conditions could be diagnosed within the same healthcare encounter, the number of diagnoses of clinical indicator conditions exceed the number of missed opportunities. MSP (Medical Services Plan); DAD (Discharge Abstract Database). Due to privacy concerns, cells with less than 5 counts cannot be further specified.
(DOCX)
