# Supplementary material for: Missed opportunities for earlier diagnosis of HIV in British Columbia, Canada: A retrospective cohort study
Source: PLoS One. 2019 Mar 21;14(3):e0214012. doi: 10.1371/journal.pone.0214012 (PMC6428302; doi:10.1371/journal.pone.0214012)
Supplement: S1 File — Additional methodological information pertaining to the data utilized in this study. (DOCX) [file pone.0214012.s001.docx]

**Methodological Supporting Information.** Additional methodological information pertaining to the data utilized in this study.

This study was carried out using data from the British Columbia Seek and Treat for Optimal Prevention of HIV/AIDS (STOP HIV/AIDS) population-based cohort, which is derived from various linkages between provincial administrative databases.

**Data Steward: British Columbia Centre for Disease Control** [1, 2]

1. Provincial HIV/AIDS Surveillance Database: a surveillance database that collates all HIV laboratory testing, new HIV diagnosis and occurrence of AIDS-defining illnesses data;

**Data Steward: British Columbia Centre for Excellence in HIV/AIDS** [3]

1. Drug Treatment and Laboratory Databases, which captures all antiretroviral dispensing data, plasma viral load testing, drug resistance testing, occurrence of AIDS-defining illnesses, approximately 85% of CD4 cell count measurements, and key patient demographic information;

**Data Steward: British Columbia Ministry of Health** [4]

1. The Medical Services Plan (MSP) billing database, which captures HIV and non-HIV-related inpatient and outpatient services provided by physicians and supplementary health care practitioners, as well as diagnostic procedures. This database also contains cost associated with claims paid through fee-for-service and the Alternative Payment Program;
2. Home and Community Care database, which captures a variety of services including hospice and home nursing care, adult day services, assisted living, respite care, residential and convalescent care
3. Mental Health Services database, which captures utilization of mental health services including fee-for-service, institutional care, community clinics and acute care.
4. Addictions Information Management Systems, which captures referral to treatment for alcohol, drug or gambling addictions.
5. The PharmaNet database is a real-time system, which captures all prescriptions for drugs and medical supplies dispensed from community pharmacies in BC as well as prescriptions dispensed from hospital outpatient pharmacies use at home. Note that this database does not capture antiretroviral dispensing data;
6. The Client Roster or Consolidation File, which captures individual demographic and geographic data. This database is also used to construct population denominators.

**Data Steward: Canadian Institute for Health Information** [5]

1. The Discharge Abstract Database (DAD), which captures all discharges, transfers and deaths of in-patients and day surgery patients from acute care hospitals across BC;

**Data Steward: British Columbia Vital Statistics Agency** [6]

1. The Vital Statistics database, which records death information of all BC’s residents.

**Rurality variable**

The rurality variable was constructed by classifying local health areas into 9 sub-categories: 1) metro-core, 2) metro-inner suburb, 3) metro-outer suburb, 4) mostly urban with large centre, 5) mostly urban with medium centre, 6) mixed urban/rural, 7) rural <1 hour from hospital, 8) rural ≥1 to <2.5 hours from hospital, and 9) rural ≥2.5 hours from hospital. These categories were then combined into the 3 broader categories, as: urban/metro (sub-categories 1-3), mixed: (sub-categories 4-6), and rural: (sub-categories 7-9). Given that full postal codes were not available due to privacy concerns, the above-mentioned definition for rurality was used as an alternative.

**References**

1. British Columbia Centre for Disease Control. HIV/AIDS Information System (HAISYS). Clinical Prevention Services, British Columbia Centre for Disease Control, 2016. Available from: <http://www.bccdc.ca/about/accountability/data-access-requests/public-health-data>.

2. British Columbia Centre for Disease Control Public Health Laboratory (2016):. HIV laboratory testing datasets (tests: ELISA, Western blot, NAAT, p24, culture). Clinical Prevention Services, British Columbia Centre for Disease Control. MOH (2016) 2016 [12 July 2018]. Available from: <http://www.bccdc.ca/about/accountability/data-access-requests/public-health-data>.

3. Patterson S, Cescon A, Samji H, Cui Z, Yip B, Lepik KJ, et al. Cohort Profile: HAART Observational Medical Evaluation and Research (HOMER) cohort. Int J Epidemiol. 2015;44(1):58-67. doi: 10.1093/ije/dyu046. PubMed PMID: 24639444; PubMed Central PMCID: PMCPMC4339756.

4. British Columbia Ministry of Health (2016):. Medical Services Plan (MSP) Payment Information File; Consolidation File (MSP Registration & Premium Billing); Home & Community Care (Continuing Care); Mental Health; PharmaNet. British Columbia Ministry of Health [publisher]. Data Extract. MOH (2016). Available from: <http://www2.gov.bc.ca/gov/content/health/conducting-health-research-evaluation/data-access-health-data-central>.

5. Canadian Institute of Health Information (2016):. Discharge Abstract Database (Hospital Separations). British Columbia Ministry of Health [publisher]. Data Extract. MOH (2016). Available from: <http://www2.gov.bc.ca/gov/content/health/conducting-health-research-evaluation/data-access-health-data-central>.

6. British Columbia Vital Statistics Agency (2016):. Vital Statistics. British Columbia Ministry of Health [publisher]. Data Extract. MOH (2016). Available from: <http://www2.gov.bc.ca/gov/content/health/conducting-health-research-evaluation/data-access-health-data-central>.
