# Supplementary material for: Missed opportunities for earlier diagnosis of HIV in British Columbia, Canada: A retrospective cohort study
Source: PLoS One. 2019 Mar 21;14(3):e0214012. doi: 10.1371/journal.pone.0214012 (PMC6428302; doi:10.1371/journal.pone.0214012)
Supplement: S1 Table — Note: ICD 9: International Classification of Diseases (Ninth Revisions); ICD 10: International Classification of Diseases (Tenth Revisions). (DOCX) [file pone.0214012.s002.docx]

**S1 Table.** List of the International Classification of Diseases (Ninth and Tenth Revisions) diagnosis codes selected for the case-finding algorithm for clinical indicator conditions.

| **Clinical Indicator Conditions** | **Disease Group** | **ICD 9 Codes** | **ICD 10 Codes** |
| --- | --- | --- | --- |
| Anal carcinoma/dysplasia | Anal Carcinoma | 230.5, 230.6, 569.44 | D01.3, K62.82 |
| Candidaemia | Mucosal fungal infections | 112.5 | B37.7 |
| Cervical dysplasis | Cervical dysplasia/cancer | 233.1, 622.1, 690.1 | D06, N87, L21 |
| Dermatitis / seborrhoeic rash | Skin disorders | 690.10 | L21.9 |
| Guillain-Barré syndrome | Peripheral nervous system diseases | 357.0 | G61.0 |
| Hep B or C (acute or chronic) | Hepatitis B or C | 070.2, 070.3, 070.41, 070.44, 070.51, 070.54, 070.7 | B16, B17.0, B17.1, B18.0, B18.1, B18.2, B19.1 |
| Herpes zoster in an individual <50 years old | Other viral infections | 053 | B02.1 |
| Invasive pneumococcal disease | Pneumoccocal diseases | 320.1, 567.1 | A40.3, G00.1, K65.0 |
| Lung carcinoma | Lung carcinoma | 231.2 | D02.2 |
| Lymphoma | Lymphoma | 200, 201, 202 | C81, C82, C83, C84, C85, C86 |
| Mononeuritis | Peripheral nervous system diseases | 354.5 | G58.7 |
| Multiple sclerosis-like disease | Central nervous system Diseases | 046.3  710.0  088.81  079.51  710.2  437.4  136.1, 711.2 | A 81.2  M32  A69.2  B97.33  M35.0  I67.7  M35.2 |
| Oral hairy leucoplakia | Leucoplakia | 528.6 | K13.3 |
| Peripheral neuropathy of  unknown origin | Peripheral nervous system diseases | 356.9 | G60.9  L40.8, L40.9 |
| Severe or atypical psoriasis | Skin disorders | 696.1 | A60 |
| Sexually transmitted infections | Sexually transmitted infections | 054.1  099.1, 099.5  091, 092, 093, 094, 095, 096, 097  098  131  795.05, 795.15, 795.09, 796.75, 796.79, 078.11 | A55, A56  A51, A52, A53  A54  A59  R85.81, R85.82, R87.81, R87.82, A63.0  F01.2 |
| Sub-cortical dementia | Central nervous system diseases | 294.1 | F01.3  F02.3  R19.7 |
| Unexplained chronic diarrhoea | Diarrhea | 787.91 | N18.9 |
| Unexplained chronic renal impairment | Renal disorders | 585.9 | D69.3, D69.49, D69.5, D69.6, D70, D72.81, M31.1 |
| Unexplained leukocytopenia/thrombocytopenia (>4 weeks) | Other blood disorders | 287.3, 287.31, 287.39, 287.5, 288.0, 288.5, 446.6 | I88, L04, R59 |
| Unexplained lymphadenopathy | Lymphatic disorders | 289.1, 289.2, 289.3, 683, 785.6 | B37.0, B37.83 |
| Unexplained oral candidiasis | Mucosal fungal infections | 112.0 | R63.4, R64 |
| Unexplained weight loss | Weight loss | 783.21, 799.4 | B55.0 |
| Visceral leishmaniasis | Protozoan infections | 085.0 | J13, J14, J15 |
| Bacterial pneumonia, recurrent | Pneumonia | 481, 482, 486 | B37.1 |
| Candidiasis of bronchi, trachea, or lungs | Mucosal fungal infections | 112.4 | B37.81 |
| Candidiasis of esophagus | Mucosal fungal infections | 112.8 | C53 |
| Cervical cancer, invasive | Cervical dysplasia/cancer | 180 | B38.3, B38.4, B38.7, B38.8, B38.9 |
| Coccidioidomycosis (disseminated or extrapulmonary) | Mucosal fungal infections | 114.1, 114.2, 114.3, 114.9 | B45.1, B45.2, B45.3, B45.7, B45.8, B45.9 |
| Cryptococcosis (extrapulmonary) | Mucosal fungal infections | 321 | A07.2 |
| Cryptosporidiosis, chronic intestinal (>1 month's duration) | Protozoan infections | 007.4 | B25.0, B25.2, B25.8, B25.9 |
| Cytomegalovirus disease (other than liver, spleen, or nodes), | Other viral infections | 484.1 | G93.4 |
| Cytomegalovirus retinitis (with loss of vision) |  |  |  |
| Encephalopathy, HIV related (dementia) | Central nervous system diseases | 348.3 | B00.2 |
| Herpes simplex: chronic ulcers (>1 month's duration) | Other viral infections | 054.2 | B39.3, B39.4, B39.5, B39.9 |
| Histoplasmosis, disseminated or extrapulmonary | Mucosal fungal infections | 115.01, 115.02, 115.03, 115.04, 115.09, 115.11, 115.12, 115.13, 115.14, 115.19, 115.91, 115.92, 115.93, 115.94, 115.99 | A07.3 |
| Isosporiasis, chronic intestinal (>1 month's duration) | Protozoan Infections | 007.2 | A07.3 |
| Kaposi Sarcoma | Kaposi Sarcoma | 176 | C46 |
| Lymphoma, Burkitt | Lymphoma | 200.2 | C83.7 |
| Lymphoma, immunoblastic (or equivalent term) | Lymphoma | 200.0 | C83.3 |
| Lymphoma, primary, of brain | Lymphoma | 200.5 | C83.39, C85.89 |
| M. avium complex or M. kansasii, disseminated or extrapulmonary | Other mycobacterial diseases | 031.1, 031.2, 031.8 | A31.1, A31.2, A31.8 |
| M. tuberculosis (disseminated or extrapulmonary) | Tuberculosis | 013, 014, 015, 016, 017, 018 | A17, A18, A19 |
| M. tuberculosis (pulmonary) | Tuberculosis | 010, 011, 012 | A15, A16 |
| Mycobacterium, other species or unidentified species | Other mycobacterial diseases | 031.8, 031.9 | A31.8, A31.9 |
| Pneumocystis carinii pneumonia | Pneumonia | 136.3 | B59 |
| Progressive multifocal leukoencephalopathy | Central nervous system diseases | 046.3 | A81.2 |
| Toxoplasmosis of brain | Central nervous system diseases | 130.0 | B58.2 |
| Wasting syndrome attributed to HIV | Weight loss | 799.4 | R64 |
| Anemia related to deficiencies and unspecified causes | Anemia | 280, 281.1, 281.2, 283.0, 283.2, 283.9, 284.9, 285.9 | D50.0, D50.8, D50.9, D51.8, D51.9, D52.0, D52.8, D52.9, D59.1, D59.8, D59.9, D61.3, D64.9 |

Note: ICD 9: International Classification of Diseases (Ninth Revisions); ICD 10: International Classification of Diseases (Tenth Revisions).
