# Supplementary material for: Missed opportunities for earlier diagnosis of HIV in British Columbia, Canada: A retrospective cohort study
Source: PLoS One. 2019 Mar 21;14(3):e0214012. doi: 10.1371/journal.pone.0214012 (PMC6428302; doi:10.1371/journal.pone.0214012)
Supplement: S2 Table — Note: Given that multiple clinical indicator conditions could be diagnosed within the same healthcare encounter, the number of diagnoses of clinical indicator conditions exceed the number of missed opportunities. MSP (Medical Services Plan); DAD (Discharge Abstract Database). Due to privacy concerns, cells with less than 5 counts cannot be further specified. (DOCX) [file pone.0214012.s003.docx]

**S2 Table.** Diagnoses of clinical indicator conditions categorized by distinct disease groups for all three analyses.

**(A)** Five-year analysis.

| **Clinical Indicator Groups** | **Total (n=660)** | **Percentage** |
| --- | --- | --- |
|  |  |  |
| Recurrent Pneumonia | 196 | 30% |
| Anemia | 114 | 18% |
| Other Viral Infections | 85 | 13% |
| Sexually Transmitted Infections | 61 | 9% |
| Lymphatic Disorders | 51 | 8% |
| Hepatitis B or C | 39 | 6% |
| Mucosal Fungal Infections | 33 | 5% |
| Lymphoma | 15 | 2% |
| Skin Disorders | 12 | 2% |
| Tuberculosis | 12 | 2% |
| Other Blood Disorders | 11 | 2% |
| PNS Diseases | 9 | 1% |
| Weight Loss | 7 | 1% |
| Autoimmune Diseases | 5 | <1% |
| Cervical Dysplasia/Cancer | <5 | <1% |
| Pneumoccocal Diseases | <5 | <1% |
| Renal Disorders | <5 | <1% |
| Anal Carcinoma | <5 | <1% |
| Diarrhoea | <5 | <1% |

**(B)** Three-year analysis.

| **Clinical Indicator Groups** | **Total (n=515)** | **Percentage** |
| --- | --- | --- |
|  |  |  |
| Recurrent Pneumonia | 157 | 31% |
| Anemia | 98 | 19% |
| Other Viral Infections | 64 | 12% |
| Sexually Transmitted Infections | 46 | 9% |
| Lymphatic Disorders | 38 | 7% |
| Mucosal Fungal Infections | 30 | 6% |
| Hepatitis B or C | 25 | 5% |
| Lymphoma | 14 | 3% |
| Skin Disorders | 12 | 2% |
| Other Blood Disorders | 9 | 2% |
| Weight Loss | 6 | 1% |
| Autoimmune Diseases | <5 | <1% |
| Cervical Dysplasia/Cancer | <5 | <1% |
| Tuberculosis | <5 | <1% |
| Pneumoccocal Diseases | <5 | <1% |
| Renal Disorders | <5 | <1% |
| Anal Carcinoma | <5 | <1% |
| Diarrhoea | <5 | <1% |
| PNS Diseases | 0 | 0% |

**(C)** One-year analysis.

| **Clinical Indicator Groups** | **Total (n=293)** | **Percentage** |
| --- | --- | --- |
|  |  |  |
| Recurrent Pneumonia | 96 | 33% |
| Anemia | 61 | 21% |
| Mucosal Fungal Infections | 27 | 9% |
| Lymphatic Disorders | 25 | 9% |
| Other Viral Infections | 22 | 8% |
| Sexually Transmitted Infections | 19 | 7% |
| Hepatitis B or C | 15 | 5% |
| Lymphoma | 6 | 2% |
| Skin Disorders | 6 | 2% |
| Other Blood Disorders | 5 | 2% |
| Weight Loss | <5 | 2% |
| Autoimmune Diseases | <5 | <2% |
| Renal Disorders | <5 | <2% |
| Cervical Dysplasia/Cancer | <5 | <2% |
| Diarrhoea | <5 | <2% |
| Tuberculosis | <5 | <2% |
| Anal Carcinoma | 0 | 0% |
| Pneumoccocal Diseases | 0 | 0% |
| PNS Diseases | 0 | 0% |

Note: Given that multiple clinical indicator conditions could be diagnosed within the same healthcare encounter, the number of diagnoses of clinical indicator conditions exceed the number of missed opportunities. MSP (Medical Services Plan); DAD (Discharge Abstract Database). Due to privacy concerns, cells with less than 5 counts cannot be further specified.
